# Systems glycobiology for discovering drug targets, biomarkers, and rational designs for glyco-immunotherapy

**DOI:** 10.1186/s12929-021-00746-2

**Published:** 2021-06-22

**Authors:** Austin W. T. Chiang, Hratch M. Baghdassarian, Benjamin P. Kellman, Bokan Bao, James T. Sorrentino, Chenguang Liang, Chih-Chung Kuo, Helen O. Masson, Nathan E. Lewis

**Affiliations:** 1 Department of Pediatrics, University of California, 9500 Gilman Drive MC 0760, La Jolla, San Diego, CA 92093 USA; 2 The Novo Nordisk Foundation Center for Biosustainability at the University of California, La Jolla, San Diego, CA 92093 USA; 3 Bioinformatics and Systems Biology Graduate Program, University of California, La Jolla, San Diego, CA 92093 USA; 4 Department of Bioengineering, University of California, La Jolla, San Diego, CA 92093 USA; 5 The National Biologics Facility, Technical University of Denmark, Kongens Lyngby, Denmark

**Keywords:** Glycosylation machinery, Cancer immunotherapy, CAR-T cell therapy, Immune checkpoint, Systems glycobiology, And Glyco-immunotherapy

## Abstract

Cancer immunotherapy has revolutionized treatment and led to an unprecedented wave of immuno-oncology research during the past two decades. In 2018, two pioneer immunotherapy innovators, Tasuku Honjo and James P. Allison, were awarded the Nobel Prize for their landmark cancer immunotherapy work regarding “cancer therapy by inhibition of negative immune regulation” –*CTLA4* and *PD-1* immune checkpoints. However, the challenge in the coming decade is to develop cancer immunotherapies that can more consistently treat various patients and cancer types. Overcoming this challenge requires a systemic understanding of the underlying interactions between immune cells, tumor cells, and immunotherapeutics. The role of aberrant glycosylation in this process, and how it influences tumor immunity and immunotherapy is beginning to emerge. Herein, we review current knowledge of miRNA-mediated regulatory mechanisms of glycosylation machinery, and how these carbohydrate moieties impact immune cell and tumor cell interactions. We discuss these insights in the context of clinical findings and provide an outlook on modulating the regulation of glycosylation to offer new therapeutic opportunities. Finally, in the coming age of systems glycobiology, we highlight how emerging technologies in systems glycobiology are enabling deeper insights into cancer immuno-oncology, helping identify novel drug targets and key biomarkers of cancer, and facilitating the rational design of glyco-immunotherapies. These hold great promise clinically in the immuno-oncology field.

**Supplementary Information:**

The online version contains supplementary material available at 10.1186/s12929-021-00746-2.

## Background

### Glycosylation in immunity and cancer

The first clinical demonstration that transformed cells can be identified as pathogenic by the immune system was recorded in the 1890s [1]. As our knowledge of immunity, immuno-oncology, and drug development has since increased, the idea of harnessing the body’s natural defenses to fight cancer (i.e., cancer immunotherapy) is now becoming a reality [2, 3]. A crucial insight into this endeavor is that engagement of immune checkpoint molecules (i.e., programmed cell death protein 1 (*PD-1*) and cytotoxic T-lymphocyte-associated antigen 4 (*CTLA4*)) is a key mechanism facilitating tumor anti-pathogenicity. In the past two decades, many cancer immunotherapies (Table 1) have been developed as promising therapeutics for this disease. However, due to myriad tumor immune evasion mechanisms [4], the efficacy of immunotherapy has remained limited, indicating that there is considerable room for improvement.

**Table 1:** FDA approved cancer immunotherapies (including immune checkpoint therapies and adoptive cell therapies)

Category	Target	Name	Indication	Source
Immune Checkpoint Therapeutic (ICI)	*PD-1* or *PD-L1*	Nivolumab (Opdivo)	Various cancers (e.g., Melanoma, Non-Small Cell Lung Cancer, etc.)	(1)
		Pembrolizumab (Keytruda)	Various cancers (e.g., Melanoma, classical Hodgkin Lymphoma, Primary Mediastinal B-cell Lymphoma, etc.)	(2)
		Atezolizumab (Tecentriq)	Non-Small Cell Lung Cancer, Urothelial Carcinoma, and Breast Cancer	(3)
		Cemiplimab (Libtayo)	Cutaneous Squamous Cell Carcinoma	(4)
		Durvalumab (Imfinzi)	Non-Small Cell Lung Cancer	(5)
		Avelumab (Bavencio)	Merkel Cell Carcinoma, and Urothelial Carcinoma	(6)
	*CTLA4*	Ipilimumab (Yervoy)	Melanoma, Renal Cancer, and MSI	(7)
Chimeric Antigen Receptor T-cell Therapy (CAR-T)	*CD19*	Axicabtagene ciloleucel (Yescarta)	Non-Hodgkin Lymphoma	(8)
		Tisagenlecleucel (Kymriah)	Non-Hodgkin Lymphoma	(9)

1. http://chemocare.com/chemotherapy/drug-info/Nivolumab.aspx

2. https://www.keytruda.com

3. https://www.tecentriq.com

4. https://www.libtayohcp.com

5. https://www.imfinzi.com

6. https://www.bavencio.com/hcp

7. https://www.yervoy.com/YervoyGateway

8. https://www.yescarta.com

9. https://www.hcp.novartis.com

Many recent publications, while in the early stages in terms of demonstrated clinical efficacy, have indicated that leveraging glycosylation can improve cancer immunotherapy and enable better treatment outcomes [5–7]. Glycosylation plays important roles in organismal development [8], cell–cell communication [9], and numerous fundamental cellular functions [10] such as translation and metabolism. Alterations in glycosylation can influence the ability of cell-surface receptors in oligomerization and influence the sensitivity of these receptor systems to stimulation. These central roles make glycosylation a hub for the pathophysiological processes in cancer [11], including tumor growth, proliferation, immunity, and metastasis. Many tumor-associated glycans on the tumor glycocalyx protect against attacks from the immune system and trigger immunosuppressive signaling through glycan-binding receptors [5]. For example, the glycan epitopes of sialylated structures, the Tn and Lewis antigens, engage with the lectin receptors, leading to distinct mechanisms of immune suppression. Importantly, aberrant tumor glycosylation creates neo-antigens that are emerging as potential targets for tumor immunotherapy.

In parallel, the structural architecture and biological function of immune checkpoint molecules may also be influenced by glycosylation. For example, N-glycans can stabilize the immune checkpoint *PD-L1* by lessening its proteasomal degradation and thereby increasing its immunosuppressive effect [12]. Apparently, glycosylation can be either a friend or foe to cancer immunotherapy, depending on the context.

### Glycan synthesis and regulation

Glycan synthesis in the complex glycosylation machinery is highly stochastic and compartmentalized. We define glycosylation machinery here by the collection of molecules (e.g., enzymes and sugar donors) and organelles (e.g., Golgi) required for the modification of proteins with carbohydrates [13]. In this context, patterns of glycan synthesis are dependent on the expression and activity of a few hundred enzymes (glycosyltransferases and glycosidases) and by the availability of precursor monosaccharides [14].

Altered glycosylation in cancer can be accounted for by epigenetic regulation, such as changes in DNA methylation and microRNA (miRNA) abundance [15]. Recently developed methods such as the miRNA proxy approach [16] have successfully identified several miRNA molecules as important regulators of tumor glycan synthesis. However, due to its regulatory complexity, comprehensively understanding the mechanisms of glycan biosynthesis remains elusive [17]. As such, there are many opportunities to further elucidate the regulatory changes that yield abnormally glycosylated molecules and consequently promote cancer immune evasion [18].

In particular, there have been few attempts to establish a holistic examination of the role of epigenetic regulation in the expression and activity of glycosylation machinery. Here we aim to describe recent research on glycosylation machinery and its associated regulatory changes in cancer immunity, which could ultimately be harnessed for rational design and clinical use of glyco-immunotherapies. We begin by discussing the current state of cancer immunotherapeutics, with a special focus on the U.S. Food and Drug Administration (FDA) approved *Immune Checkpoint Therapeutics* and *Adoptive Cell Immuno-Therapies* (Table 1). Next, we examine recent knowledge on how glycosylation modulates these immunotherapies, focusing on glycan biosynthesis and nucleotide sugar synthesis pathways. We also explore how glycan synthesis has been epigenetically dysregulated by microRNAs to generate neo-antigens on tumor cells. Lastly, we highlight how latest systems glycobiology tools and analytical methods may address existing knowledge gaps in the interplay between glycosylation, regulation, and cancer immunity; these approaches can facilitate the development of the next generation of glyco-immunotherapies.

## Current knowledge of how glycosylation effects interactions between immune checkpoint therapies and their targets

**Fig. 1:**
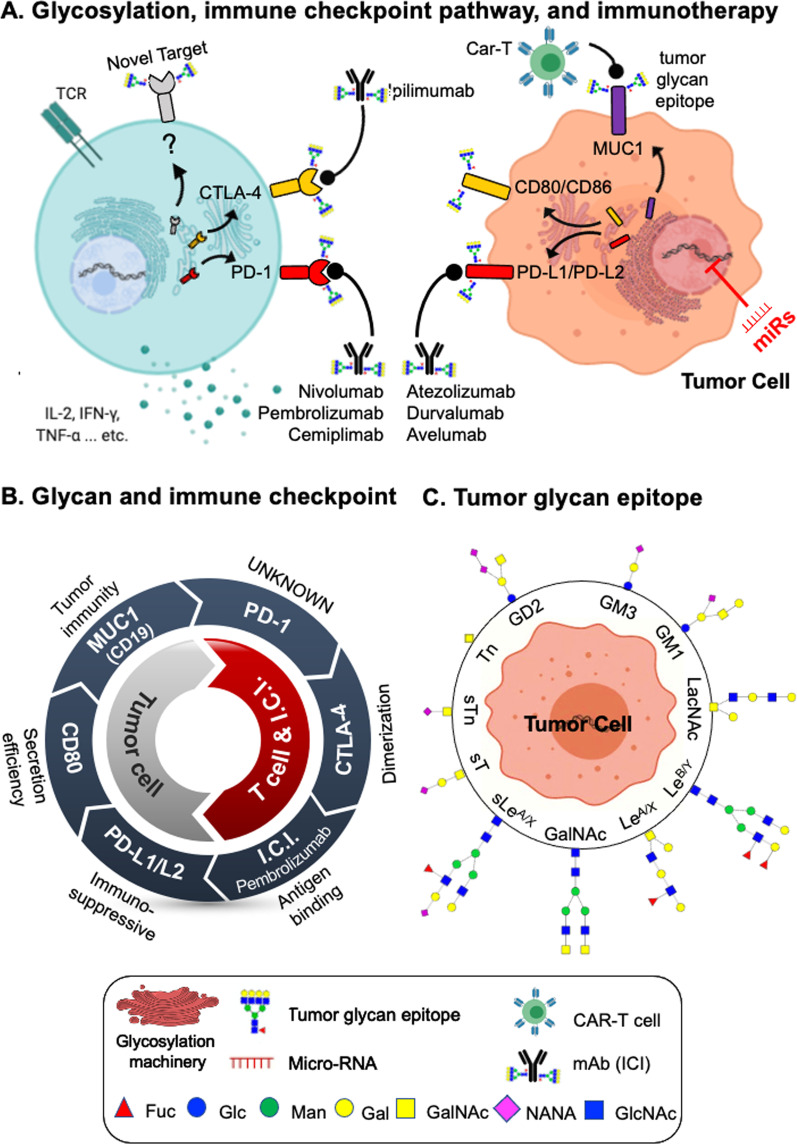
Current knowledge about the glycosylation roles in the cancer immunotherapy. **A** Schematic view of the glycosylation, cancer immunotherapies (mAb-based ICIs and CAR-T cell), and their targets. Cancer immunotherapies are developed to target the immune checkpoints (e.g., *PD-1* and *CTLA-4* on the T cell or their ligands (e.g., PD-L1/PD-L2 and CD80/CD86) on the tumor cell), which are processed via the glycosylation machinery and decorated with glycans. The glycosylation machinery is regulated by miRNAs (red color). These glycans might impact on the efficacy of immune checkpoints therapies. **B** Current knowledge about glycosylation on the immune checkpoint pathway: tumor cell (*MUC1*, *CD80*, and *PD-L1*/*L2*), T cell (*PD-1* and *CATLA4*), and immune checkpoint therapeutic (ICI). **C** 11 well-known glycan targets (tumor glycan epitopes) of cancer immunotherapeutic on the tumor cells

A whole suite of therapeutics—immune checkpoint inhibitors (ICIs), cell therapies, and vaccines—meant to enhance anti-tumor immunity and counteract tumor escape is at various stages of development. Table 1 provides examples of several therapeutics and their targets. This includes seven ICIs and two chimeric antigen receptor T-cells (CAR-T) approved by the FDA [19]. Interested readers are encouraged to refer to supplement (Additional file 1: Appendices A–D) on the molecular mechanisms of these cancer immunotherapies. Glycosylation can impact the affinity of intercellular protein–protein interactions, and thus downstream signaling of membrane protein receptors (Fig. 1A). These membrane proteins are processed via the glycosylation machinery and interact with a number of enzymes that add post-translational modifications, e.g., N-linked glycosylation. These glycosylation patterns, much like ligands of immune inhibitory receptors, can engage glycan binding receptors and, in the context of cancer, diminish the immune response. Interactions between glycans and immune inhibitory receptors thus provide many potential targets for engineering ligand-receptor binding affinity. However, while many tumor-associated glycan epitopes (Fig. 1C) have been identified and are currently being evaluated for potential clinical applications [20], glycosylation of immune checkpoint therapies and their targets has largely been overlooked. Below, we review current knowledge regarding the effects of glycosylation on immune checkpoints (Fig. 1B).

### Impacts of glycosylation on the immune checkpoint therapeutic and PD-1 interactions

Nivolumab and pembrolizumab are two FDA-approved therapeutics targeting *PD-1* (Table 1). Studies have demonstrated the inability of nivolumab to bind a non-glycosylated form of *PD-1* [21]. Yet, attempts to find a specific, mechanistic explanation for this observation have not succeeded. For example, Tan et al. [22] evaluated the effect of *PD-1* glycosylation on the interaction with nivolumab, but their results demonstrated that none of the N-linked glycosylation sites are necessary for binding with nivolumab. Other efforts have explored how glycosylation of therapeutics effects their interactions with *PD-1*. For example, Scapin et al. [23] demonstrated that glycosylation of the CH2 domain of IgG4 pembrolizumab causes a 120° conformational rotation, resulting in the attached N-linked glycan having a higher exposure to solvent relative to other IgG subclasses and likely reducing its affinity to Fc receptors and complement C1q. Their study demonstrated that, while the underlying mechanisms are unknown, solvent exposure implies that this glycan plays a role in pembrolizumab-*PD-1* binding interactions. The limited knowledge with regard to whether glycosylation has a functional role in these binding interactions and the underlying mechanisms by which glycans may mediate these interactions highlight the need for additional research in this area.

### Impacts of glycosylation on the immune checkpoint therapeutic and PD-L1 interactions

Glycosylation of *PD-L1* at N192, N200, and N219 in cancer cells is proven to prevent its degradation, enhancing its immunosuppressive properties [12]. Furthermore, inhibiting an upstream mechanism of glycan stabilization enhanced the efficacy of *PD-1* blockade, signifying the potential of targeting the biosynthetic enzymes that modulate glycosylation. In a separate study, Wang et al. [24] found that treatment with tunicamycin, which inhibits N-linked glycosylation, substantially reduced the expression of *PD-L2* in colorectal cancer. Recently, Li et al. [25] successfully generated a monoclonal antibody (mAb) for targeting glycosylated *PD-L1* in triple negative breast cancer (TNBC) cells. This mAb blocks associations between *PD-L1* and *PD-1*, leading to enhanced internalization and degradation of *PD-L1* and highly effective eradication of TNBC tumors. Glycosylation of *PD-L1* has been demonstrated to be required for *PD-1* interaction through Gal-beta1-4GlcNAc (LacNAc) glycosylation mediated by a glycosyltransferase B3GNT3 [25]. Moreover, deglycosylated *PD-L1* has been demonstrated to be a better biomarker to guide immunotherapy [26]. Altogether, targeting glycosylated *PD-L1* holds great promise to be served as a means to improve immunotherapy response [27].

### Impacts of glycosylation on CTLA-4 and CD80 interactions

Despite extensive studies to understand *CTLA-4*, their binding partners *CD80*/*CD86*, and the therapeutics developed to target them, there has been little focus on potential therapeutic avenues related to their glycosylation patterns. Nonetheless, studies have shown that dimerization of *CTLA-4* via disulfide bonds enhances its surface expression, perhaps improving efficiency of secretion. Glycosylation, in conjunction with disulfide bond formation, is necessary for this dimerization to occur [28]. Furthermore, while non-glycosylated *CD80* can bind *CTLA-4*, it demonstrates a decrease in overall expression levels, likely due to decreased secretory efficiency. Interestingly, binding of non-glycosylated *CD80* to *CTLA-4* is functionally equivalent to antagonistic blockade [29].

## Glycosylation machinery and its regulatory mechanism in tumor cells

**Fig. 2:**
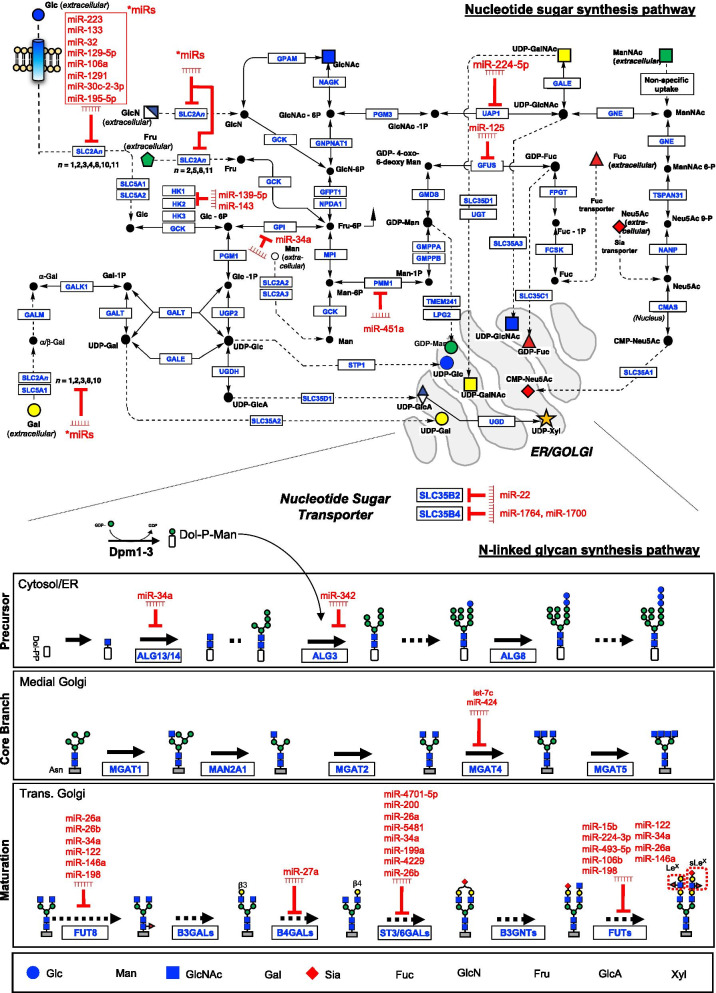
Glycan synthesis and epigenetic miRNA-regulation of the glycosylation machinery in the tumor microenvironment. (Top panel) MiRNA regulation in the glycan precursor synthesis (sugar/nucleotide sugar transport and monosaccharide synthesis). Sugar transporters transport different types of extracellular sugars into cells (dashed lines), and the sugars are further converted into nucleotide sugars (solid lines). The filled black circle indicated metabolites leading to nucleotide sugars, and all the other graphical symbols match those in Symbol Nomenclature for Glycans (SNFG) (https://www.ncbi.nlm.nih.gov/glycans/snfg.html). The nucleotide sugar synthesis pathway is replotted from [96]. (Bottom panel) MiRNA regulation in the *N*-linked glycan synthesis. The monosaccharides will be transported (dashed lines) to ER or Golgi, in which a variety of glycosyltransferases are responsible for a series of reactions (e.g., precursor synthesis, core branching, and maturation; indicated in the bottom panel) to synthesize complex glycans. All the miRNA regulations in the glycosylation machinery are indicated by red colors, in which the miRNAs were experimentally validated to target these glycosyltransferases (see details in the main text). All the enzymes or transporters are indicated by their gene symbols (blue colors)

Increasing evidence indicates that tumor-associated glycans play an essential role during malignancy by impacting many biological processes involved in the cell transformation process, including tumor angiogenesis, intracellular and intercellular signaling, immune regulation, tumor matrix interactions, and metastasis; these alterations impact tumor development and pharmaceutical efficacy [5, 30]. Aberrant tumor-specific glycosylation is the result of alterations in glycan biosynthetic pathways. Glycan biosynthesis pathways involve multiple steps, and changes to any of these steps can yield unexpected changes in a cell’s glycan repertoire. Thus, there is a need to further unravel the regulation of each enzymatic step in glycan synthesis. Here we review current knowledge on which miRNAs affect tumor-associated glycan epitopes by dysregulating glycosylation machinery. Specifically, we focus on the role of miRNA regulation in glycan precursor synthesis (sugar/nucleotide sugar transport and monosaccharide synthesis) and *N*-linked glycan synthesis (Fig. 2). Interested readers are encouraged to refer to two recent review papers on the other types of glycosylation [15, 31].

In the proceeding section, we discuss how advances in the field of glycobiology, which have not yet been translated to immuno-oncology, may address existing mechanistic knowledge gaps in these regulatory interactions. Several glycosyltransferases regulated by miRNAs which result in altered glycan epitopes (Fig. 1C) across different cancers are summarized in Table 2. For example, alpha-2,8-sialytransferase 1 (*ST8SIA1*) and beta-1,4-N-acetyl-galactosaminyltransferase 1 (*B4GALNT1*), regulated by miR-33a and let-7e, increase expression of gangliosides (GD2 and GD3) in ovarian cancer [32].

**Table 2:** miRNA regulation in the glycan epitope formation

miRNA	Target glycogene	Glycan epitope	Cancer	References
miR-33a; let-7e	*ST8SIA1; B4GALNT1*	GD2; GD3	Ovarian cancer	[32]
miR-199	*GCNT2*	blood group I antigen	Colon cancer	[97]
miR-200 family	*ST3GAL5*	GM3	Mesenchymal-to-Epithelial Transition (MET)	[16]
miR-9	*GALNTs*	Tn- and sTn-antigen	Various cancers	[98]
UNKNOWN	*ST3GAL1/3/4*	Selectin-binding glycans	Colon cancer	[51]
miR-34a; miR-122; miR-198	*FUT8*	Fucose	Various cancers	[44–47]

### Sugar transport and monosaccharide synthesis

Dysregulation of cell surface glucose transporters by miRNAs have been associated with changes to glucose uptake and subsequent metabolism [33]. Multiple miRNAs directly or indirectly regulate glucose transport to facilitate the unique glucose metabolism seen in various cancer types (Fig. 2; top panel). For example, a three-miRNA cluster (miR-23a, miR-27a and miR-24) of HIF1α induced miRNA moieties promotes colorectal cancer progression via remodeling of the glucose metabolic network [34]. Various other examples of miRNAs regulating the families of glucose transporters *SLC2* or *SLC45* have been outlined in Table 3. Intrinsic to glucose metabolism is the phosphorylation of the primary hexose by hexokinase to form the prominent metabolite glucose-6-phosphate. Glucose-6-phosphate is the primary precursor metabolite in the production of sugar nucleotides via the nucleotide sugar metabolic pathway [35]. Since hexokinase plays a vital role in the fate of glucose it is no surprise that changes in the expression of *HK1* and *HK2* have been linked to cancer phenotypes [36]. In the exploration of new targets for hepatocellular carcinoma, miR-139-5p was discovered to regulate the expression of *HK1* through directly targeting the transcription factor *ETS1* [37]. In a more general sense, *HK2* is seen to be abundantly overexpressed in a variety of human tumor types. It has been shown that miR-143 inhibits *HK2* expression and thus influences cancer metabolism [38].

**Table 3:** miRNA regulation in the glycan precursor synthesis

microRNA	Gene target	Function role of gene target in glycosylation	Regulatory effect in tumor	References
miR-1291	*SLC2A1*	Glucose transporter	Tumor Suppressive	[99]
miR-30c-2-3p	*SLC2A1*	Glucose transporter	Unknown	[100]
miR‐195‐5p	*SLC2A3*	Glucose transporter	Tumor Suppressive	[101]
miR-106a	*SLC2A3*	Glucose transporter	Tumor Suppressive	[102]
miR-129-5p	*SLC2A3*	Glucose transporter	Tumor Suppressive	[103]
miR-223	*SLC2A4*	Glucose transporter	Unknown	[104]
miR-133	*SLC2A4*	Glucose transporter	Unknown	[105]
miR-22	*SLC35B2*	Nucleotide Sugar Transport	Tumor Suppressive	[106]
miR-1764, miR-1700	*SLC35B4*	Nucleotide Sugar Transport	Unknown	[107]
miR-369-3p	*SLC35F5*	Nucleotide Sugar Transport	Tumor Suppressive	[108]
miR-32	*SLC45A3*	Glucose transporter	Unknown	[109]
miR-139-5p	*HK1*	Nucleotide Sugar Metabolism	Tumor Suppressive	[37]
miR-143	*HK2*	Nucleotide Sugar Metabolism	Unknown	[38]
miR-34a	*GPI*	Nucleotide Sugar Metabolism	Unknown	[40]
miR‑224‑5p	*UAP1*	Nucleotide Sugar Metabolism	Tumor Suppressive	[41]
miR‑451a	*PMM2*	Nucleotide Sugar Metabolism	Tumor Suppressive	[110]
miR‑125a-5p, miR-125b	*TSTA3*	Nucleotide Sugar Metabolism	Tumor Suppressive	[111]
miR-29a-3p, miR-29b-3p	*CMAHP*	Nucleotide Sugar Metabolism	Tumor Suppressive	[112]

The nucleotide sugar metabolic pathway produces monosaccharides for glycosylation [39], bridging glycolysis to other cellular pathways. Since glycan extension and branching is nutrient sensitive, changes in metabolic flux through the hexosamine biosynthetic pathway could impact stabilization, recruitment, and retention of the cell surface receptors by mediating the engagement of N-glycans and galectin-3. It is not fully understood how miRNAs associated with enzymes in the nucleotide sugar metabolic pathway shift the abundance of nucleotide sugar donors (NSDs) in cancer cells, but additional characterization could provide insights to novel glycan biomarkers. For example, miR-34a is capable of repressing *HK1*, *HK2*, and glucose-6-phosphate isomerase (*GPI*). *GPI* is a key enzyme related to the production of the NSD GDP-Mannose [40]. Additionally, in silico analysis reveals that the acetylhexosamine (UDP-GlcNAc, UDP-GalNAc) producing enzyme, *UAP1*, may be repressed by miR‑224‑5p; the downregulation of miR‑224‑5p may contribute to altered glycosylation in prostate cancer [41].

### N-linked glycan synthesis

Figure 2 (bottom panel) depicts miRNA dysregulation of the four major N-linked glycan synthesis processes in cancer: *Branching*, *Extension*, *Termination*, and *Decoration*. *Branching* (*Bisecting GlcNAc)*: miR-424 can inhibit *MGAT4*-mediated transfer of GlcNAc to the β1,4 linkage in *N*-linked glycans in mammary epithelium, leading to the arrest of cell cycle through *CCND1* down-regulation [42]. *Extension (Galactose)*: miR-27a has been reported to up-regulate *B4GALT3*, leading to tumorigenesis of cervical cancer [43]. *Decoration (Core Fucosylation)*: the expression of fucosyltransferase 8 (*FUT8*) can increase invasion, proliferation, metastasis, and tumor growth in many different cancers [44]. MiR-198 modulates *FUT8* expression at the level of both mRNA and protein, resulting in an invasive phenotype of colorectal cancer [45]. Moreover, several micro-RNAs (e.g., miR-122 and miR-34a) have been implicated in *FUT8* down-regulation in liver cancer [46]. Interestingly, miR-34a has been reported to exert its effect only at a translational level in hepatocarcinoma cells but not at a transcriptional level [47]. *Termination (Sialic Acid)*: miR-4701-5p down-regulates *ST3GAL1* in multi-drug resistant chronic myeloid leukemia cells [48]. The up-regulation of *ST3GAL6* is regulated by miR-26a, resulting in increased invasion in hepatocarcinoma [49]. miR-4299 was reported to silence *ST6GALNAC4,* resulting in enhanced invasive properties of human follicular thyroid carcinoma [50].

There are many additional enzymes contributing to cancer specific glycans, but how and whether miRNAs regulate their activity remains largely unknown. For example, the alpha-2,3-sialytransferases (*ST3Gal-1/-3/-4*) help synthesize selectin-binding glycans in cancer cells that contribute to hematogenous metastasis [51]. These are all potential therapeutic targets, and future directions should address improving our mechanistic understandings of aberrant tumor-glycan synthesis and associated regulatory changes.

## Systems glycobiology: emerging opportunities and challenges for the next generation of cancer immunotherapy

**Fig. 3:**
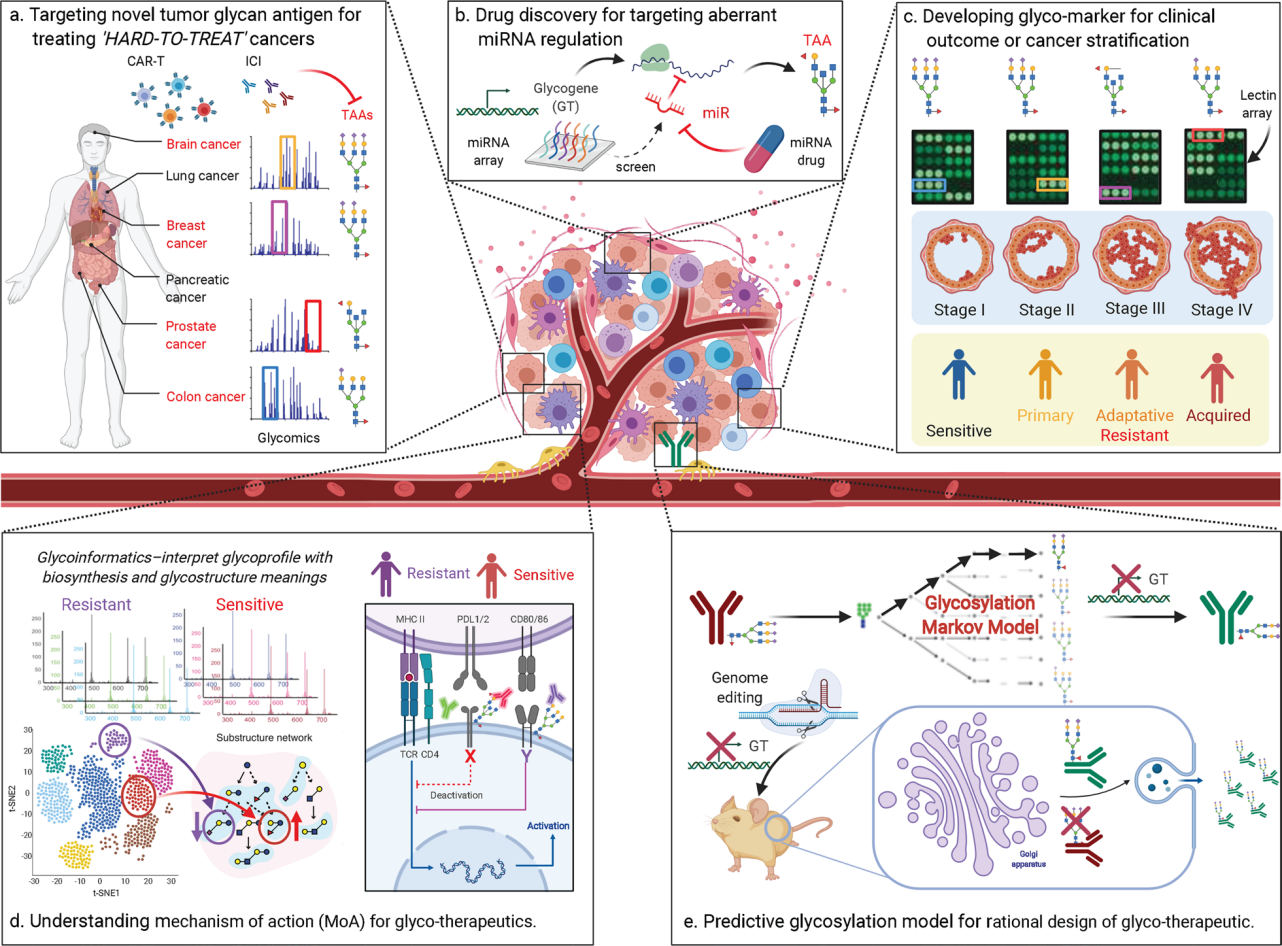
Systems glycobiology and cancer immunotherapy. **A** Targeting novel tumor glycan antigens for treating ‘*hard-to-treat*’ cancers. Systems glycobiology investigates and characterizes complex glycosylation machinery based on glycomic data, in which the altered glycan biosynthetic pathways and their generated TAAs can increase the list of potential targets for many ‘*hard-to-treat*’ cancers (e.g., prostate and brain cancers). **B** Drug discovery for targeting aberrant miRNA regulation of tumor glycans. The recently developed computational tools/databases (Table 4) and mathematical models (Sect. “Predictive glycosylation modeling for guiding rational design of immunotherapy”) for glycobiology can be used to screen glycogenes leading to aberrant glycan synthesis in cancer. By integrating with miRNA array data, the identified glycogenes could be further used to interrogate possible miRNA regulators. **C**–**D** Developing glyco-marker for clinical outcome or cancer stratification. High throughput glycomic data (including lectin array data) can aid in the discovery of novel carbohydrate biomarkers in cancer stratification and clinical outcomes. Additionally, glycoinformatics tools have facilitated analysis of glycan epitopes by deconvolving glycans from high throughput datasets into their epitopes. By integrating with recent single-cell technologies, we are able to associate them with cancer heterogeneity. All these advanced technologies hold great promise to help us gain a more comprehensive understand of mechanisms of action (MoA) for glyco-therapeutics. **E** Predictive glycosylation model for rational design of glyco-therapeutic. By mapping glycoprofiles to their respective biosynthetic enzymes and pathways, systems modeling approaches can reveal mechanisms-of-action relating glycoproteins to their associated glycosylation machinery and regulatory network, guiding rational design of immunotherapies. This figure was created with https://biorender.com

Thanks to the advents in a range of supporting ‘bridge’ technologies, the fields of systems glycobiology and cancer immunology are equipped to resolve challenging cancer immunotherapy problems (Additional file 1: Figure S1, Appendix E). In this field of ‘*Systems Glycobiology*’, novel innovations and methods are available to engineers and scientists to improve the clinical translation of glycobiology. In the following text, we discuss known and future applications of systems glycobiology to cancer immunotherapy (Fig. 3).

### Discovery of novel glycan targets for ‘hard-to-treat’ cancers

To improve the efficacy and minimize the toxicity of immunotherapies, it is critical to identify appropriate targets. The majority of current immunotherapiestarget tumor associated protein epitopes. Altered glycan biosynthetic pathways and their associated products can increase the list of potential targets [52]. This is important in cases where the list of tumor-associated protein epitopes is limited, such as solid tumor cells [53]. For example, prostate and brain cancers are resistant to checkpoint immunotherapy and have been classified as “hard-to-treat”.

We summarize four recently reviewed promising glycan targeting CARs (Fig. 1C) [54]. *First, the tumor-associated glycoprotein 72 (TAG72).* TAG72 is a truncated sTn O-glycan hapten that is widely expressed on solid tumors (e.g., endometrial and colorectal cancer). CAR-T cells have been reported to target TAG72 in gastrointestinal tumor cell lines [55] and in metastatic colorectal cancer patients [56]. *Second, the Lewis y (Le*^*y*^*).* The difucosylated carbohydrate antigen–Le^y^ is expressed in myeloid cell malignancies and epithelial derived tumors. CAR-T cells exhibit better reactivity for high Le^y^ expressing tumor cell lines [57]. *Third, the disialoganglioside glycoconjugate (GD2).* GD2 expresses on neural crest derived tumors. Recently, an anti-GD2 antibody (dinutuximab; approved by FDA) was developed for the treatment of high-risk neuroblastoma [58]. CAR-T therapy has been used in treating patients with high-risk neuroblastoma, which resulted in tumor necrosis [59]. Recently, advances in CAR-T therapeutics targeting GD2 have reported no on-target, off-tumor toxicity [60]. *Fourth, the glyco-peptide (Tn-MUC1).* Tn-MUC1 is expressed in many tumors, including ovarian, lung, prostate, and breast cancer. CAR-T cells targeting Tn-MUC1 eliminate pancreatic cancer and leukemia in xenograft models [61]. Despite these encouraging results, no glycan-targeting CAR-T cells have passed clinical phase trials yet. Of note, there are currently 10 active phase I/II MUC1 trials. These results suggest that tumor-specific glycan epitopes could offer great promise to overcome the paucity of cancer-specific targets associated with solid tumors.

Systems glycobiology aims to investigate and characterize complex glycosylation machinery based on integrating multiple omics data types [62]. The application of high-throughput approaches can significantly facilitate the discovery and characterization of tumor glycan antigens. Specifically, recent advancements in mass spectrometry (MS)-based glycomics techniques enable us to qualitatively and quantitatively study the glycome [63–65]. Table 4 summarizes recent computational tools to manage large quantities of glycoprofiling data, and several databases to aid in the interpretation of these data. However, the analysis of glycomic data remains difficult due to high glycoform heterogeneity, potential linkage ambiguity, and the costly LC–MS pipeline [66]. Future refinement of approaches to address these challenges will allow us to better comprehend glycosylation patterns in tumors. More recently, lectin microarray technologies have emerged as another important analytical approach providing a rapid analysis of glycan epitopes [67]. Specifically, lectin microarrays are a powerful technology that can directly observe the *N*-, *O*-linked and glycolipid glycomes simultaneously.

**Table 4:** Recently developed computational tools and database for glycobiology

Tool	URL	Description
GlyTouCan	https://glytoucan.org/	A comprehensive glycan structure repository
GlyGen	https://glygen.org/	A project for carbohydrate and glycoconjugate related data integration and dissemination, to retrieve information from various data sources, to integrate and harmonize this data through a user-friendly Web interface
UniCarbKB	http://www.unicarbkb.org/	A knowledge base with curated glycoconjugate information and their annotations
UniCarb-DB	https://unicarb-db.expasy.org/	A database with the structural and experimental MS-glycomic data
Glynsight	https://glycoproteome.expasy.org/glynsight/	A comparison tool that visualizes and interactively compares glycoprofiles uploaded by users. Initially, the tool was created specifically for IgG *N*-glycan profiles, but it can be extended to any data profiling *N*- or *O*-linked glycans
EpitopeXtractor	https://glycoproteome.expasy.org/epextractor/	A collection of glyco-epitopes from four sources. EpitopeXtractor helps you (1) extract all the epitopes contained in one or more glycan structures from a glycomic sample and (2) map the results in Glycdin' our epitope network viewer
GlyCreSoft	https://mobiusklein.github.io/glycresoft/docs/_build/html/	A glycan composition assigning tools for LC–MS and LC–MS/MS data that uses information on the biosynthetic network relationships among glycans
Glypy	https://github.com/mobiusklein/glypy	A well-documented glycan analysis and glycoinformatics library for Python

### Challenges and suggestions for the discovery of novel therapeutic microRNA targets of tumor glycans

Despite recent advances that have made miRNA a fascinating subject in cancer glycobiology research [68], there are several limitations that need to be addressed before achieving its full potential. A key obstacle for miRNA therapeutics is the identification of ideal miRNA targets for different types of cancers. We still lack effective experimental methods for high throughput identification of miRNAs and their regulated genes [31]. Although many computational tools have been developed to identify miRNA target genes based on transcriptomic data in the past decades [69], these methods are beset with high false positive and false negative rates. This issue is exacerbated in tools predicting miRNA targets of glycosylation-related genes [31], as many glycosylation-related genes are lowly expressed. Transcriptomics alone cannot resolve this issue since it does not accurately reflect protein abundance, especially of proteins processed via the secretory pathway [70]. Another major challenge for miRNA therapeutics is the mitigation of off-target effects [71]. Since hundreds of genes can be regulated by a single miRNA, miRNA therapeutics (miRNA mimics and miRNA inhibitors) mediated silencing of a given miRNA might lead to multiple dysregulated biological processes, resulting in toxicity and provoking unwanted clinical outcomes [72].

In light of these concerns, it is crucial to assess mechanistic functions of candidate miRNAs for guiding future endeavors in miRNA therapeutics. We foresee that systems glycobiology will make an important contribution to the refinement of miRNA prediction tools. Specifically, the aforementioned computational tools (Table 4) and glycosylation models (see examples in Sect. “Predictive glycosylation modeling for guiding rational design of immunotherapy”) can be used to screen glycogenes leading to aberrant glycan synthesis in cancer. The identified glycogenes could be further used to interrogate possible miRNA regulators by integrating with high throughput omics data. Such approaches require further development of computational tools that identify and characterize key miRNAs that both are differentially expressed and correlated with phenotypic changes in the transformed cells. Ultimately, advanced systems glycobiology techniques can map out the miRNA regulatory network modulating aberrant glycan biosynthesis and inducing phenotypic changes of the transformed cells.

### Discovery of novel glyco-markers for clinical outcomes and cancer stratification

While several experimental and FDA-approved carbohydrate biomarkers have been discovered (e.g., CA19-9 for monitoring pancreatic cancer, CA125 for monitoring ovarian cancer, and CA15-3 and CA27-29 for monitoring breast cancer; further details and examples can be found in the review by Ludwig et al. [73]), the discovery process is not only laborious but also time-consuming. Another challenge is that tumor-associated carbohydrate antigens (TACAs) are rarely unique to cancer and may be expressed at low levels on normal tissues, in which TACAs often represent incomplete biosynthetic product [74]. Thus, the "on-target off-tumor effect" of therapeutic antibodies and CAR-Ts will remain a major challenge in cancer immunotherapy, even when TACAs are targeted. As the field continues to evolve, additional types of high throughput glycomics data can provide valuable, comprehensive cellular information to aid in the discovery of novel carbohydrate biomarkers in cancer stratification and clinical outcomes. For example, rapid advances in glycomics and glycoproteomics are helping identify aberrantly glycosylated glycoproteins as biomarkers in the diagnosis and stratification of cancer types [75].

Additionally, recent advances in single-cell technologies have shed light on cancer heterogeneity [76]. While substantial single-cell studies performed on the genome [77], transcriptome [78] and proteome [79] show heterogeneous phenotypes across individual cells, progress in single-cell glycomic research has considerably lagged behind. Thus, there is a need for high-throughput and low-cost single-cell glycomics methods [80].

Recent advances in bioinformatics tools have also facilitated analysis of glycan epitopes by deconvolving glycans from high throughput datasets into their epitopes. For example, Rademacher and Paulson [81] developed a glycan fingerprinting method for studying glycan substructure diversity in glycan databases. Hosoda et al. [82] further developed a glycan multi-alignment tool to identify shared structures across glycans. EpitopeXtractor decomposes glycans into substructures; Jaiman et al. [83] recently used this substructure information to infer glycan synthesis operation. Sharapov et al. [84] made a major step towards substructure-level examination in a genome wide association study (GWAS) that examined several select substructures of blood serum *N*-glycans. Bao et al*.* [85] recently developed *GlyCompare*, a method enabling the rapid analysis and comparison of large sets of glycoprofiles by decomposing each sample into its glycan substructures.

While these tools facilitate the analysis of glycosylation data by accounting for glycan structures, the methods are typically tailored to individual types of glycosylation. In the future, biomarkers may be categorized on the basis of different omics types and personalized clinical information. Furthermore, glycomics, in conjunction with other omics data types, can provide mechanistic insights to cancer and immune cell function. These analyses will produce a comprehensive map of molecular pathways activated during tumor pathogenesis and treatment. Therefore, systems glycobiology may more precisely stratify immune-related diseases and inform personalized treatments.

### Predictive glycosylation modeling for guiding rational design of immunotherapy

Systems modeling approaches can reveal mechanisms-of-action relating glycoproteins to their associated glycosylation machinery and regulatory network, guiding rational design of immunotherapies. Many emerging methods can map glycoprofiles to their respective biosynthetic enzymes and pathways, which can address the seemingly random nature of glycan biosynthesis and degradation in the ER and Golgi [86]. Computational models of glycosylation have been under development for more than two decades [17]. In 1997, the first in silico glycosylation model [87] was developed to computationally predict glycopattern changes of 33 N-linked glycans based on expression levels and differential localization of glycosyltransferases. Many theoretical models have been established to model glycosylation at the glycan or epitope level over the last two decades (Additional file 1: Figure S1, Appendix E). However, most of these glycosylation models require a substantial number of kinetic parameters [88]. Recently, a low-parameter Markov chain method [89] has been successfully employed in modelling N-linked glycosylation. Each glycan state is modeled by a transition probability representing the stochastic transition from one glycan to the next. This model can reproduce distributions of various glycoforms and does not need detailed kinetic parameter information. This modeling framework has been used to predict how a cell line can be engineered in biosimilar design [89]. In another study, an N-linked glycosylation model [90] of Chinese hamster ovary (CHO) was developed that includes all CHO N-glycosylation genes, as well as metabolic genes related to nucleotide sugar synthesis, transport, and glycosylation. This model uses reaction flux flow stoichiometry, discrete variable state parameters, and mass balances to estimate the possible glycosylation patterns of therapeutic protein. In summary, these tools have been developed to aid in glycan annotation or modeling of glycan synthesis and are beginning to contribute to the investigations of underlying mechanisms of aberrant cancer glycosylation.

Models of glycosylation can be used for diverse applications such as studying aberrant glycosylation in cancer glycoprofiles [91] and also enabling data-driven decision making in many phases of drug discovery and development [92, 93]. They can also be used to predict harmful glycans on cancer biotherapeutics and develop methods to ensure safety and potency [94]. For example, glycosylation models can successfully predict glycoprofiles of several different glycoengineered therapeutics (e.g., Rituximab) produced in CHO cells [89, 95].

Developing models to understand how various biological layers (e.g., DNA, RNA, and protein) interact with and regulate glycosylation machinery in the context of cancer immunology will be important for future analyses. We highlight several intriguing biological questions for future research: (1) Which glycosylation machinery are activated or inhibited in tumors, and how does this altered activity impact glycosylation patterns? (2) Can we develop a predictive glycosylation model to explain the impact of glycosylation on protein features which effect therapeutic potential? (3) How can we improve tools to identify upstream regulators of glycosylation machinery that influence the ability to synthesize specific glycans? Ultimately, addressing these questions will help develop effective, safe, and affordable glycosylated immunotherapies.

## Conclusions

We propose that the adaptation of systems glycobiology tools to immune-oncology, and in particular the regulation of glycan biosynthetic pathways, will play a key role in addressing the current challenges faced by immuno-oncology. Here we surveyed recent advances in this field, and we identified knowledge gaps and opportunities for future research. The highlighted cutting-edge technologies available in systems glycobiology enable more significant insights into cancer immuno-oncology, assist in discovering novel drug targets and critical biomarkers of cancer, and facilitate the rational design of immunotherapies.

## Supplementary Information

**Additional file 1: Appendix–A.** Molecular mechanisms of cancer immunotherapies. **Appendix–B.** Novel targets to overcome tumor evasion. **Appendix–C.** Novel technologies to overcome tumor evasion. **Appendix–D.** Novel technologies to overcome graft-versus-host-disease. **Appendix–E.** The coming age of Systems Glycobiology in cancer research. **Figure S1.** The coming age of Systems Glycobiology in cancer research. (Top panel) Timeline of Nobel Prize or Milestone of cancer immunology (blue colors) and the FDA approved cancer immunotherapies (red colors). (Bottom panel) Timeline of systematic modelling of glycosylation machinery (purple colors) and the analytical methods and computational tool for study glycan epitopes (green colors).

## Data Availability

All data generated or analyzed during this study are included in this published article and references.
