# Supplementary material for: Systems glycobiology for discovering drug targets, biomarkers, and rational designs for glyco-immunotherapy
Source: J Biomed Sci. 2021 Jun 22;28:50. doi: 10.1186/s12929-021-00746-2 (PMC8218521; doi:10.1186/s12929-021-00746-2)
Supplement: Supplementary file 1 — Additional file 1: Appendix–A. Molecular mechanisms of cancer immunotherapies. Appendix–B. Novel targets to overcome tumor evasion. Appendix–C. Novel technologies to overcome tumor evasion. Appendix–D. Novel technologies to overcome graft-versus-host-disease. Appendix–E. The coming age of Systems Glycobiology in cancer research. Figure S1. The coming age of Systems Glycobiology in cancer research. (Top panel) Timeline of Nobel Prize or Milestone of cancer immunology (blue colors) and the FDA approved cancer immunotherapies (red colors). (Bottom panel) Timeline of systematic modelling of glycosylation machinery (purple colors) and the analytical methods and computational tool for study glycan epitopes (green colors). [file 12929_2021_746_MOESM1_ESM.pdf]

Systems glycobiology for discovering drug targets, biomarkers, and rational designs for glyco-immunotherapy

Austin W.T. Chiang[1,2,+,*], Hratch M. Baghdassarian[1,2,3,+], Benjamin P. Kellman[1,2,3], Bokan Bao[1,2,3], James T. Sorrentino[1,2,3], Chenguang Liang[1,4], Chih-Chung Kuo[1,2,4], Helen O. Masson[1,4], and Nathan E. Lewis[1,2,4,5]

[1] Department of Pediatrics, University of California, San Diego, La Jolla, CA 92093, USA
[2] The Novo Nordisk Foundation Center for Biosustainability at the University of California, San Diego, La Jolla, CA 92093, USA
[3] Bioinformatics and Systems Biology Graduate Program, University of California, San Diego, La Jolla, CA 92093, USA
[4] Department of Bioengineering, University of California, San Diego, La Jolla, CA 92093, USA
[5] The National Biologics Facility, Technical University of Denmark

[+] These authors contributed equally to this work

[*] Corresponding author:

Name: Austin Wan Tien Chiang
Address: 9500 Gilman Drive MC 0760, La Jolla, CA 92093
E-mail: w3chiang@health.ucsd.edu

Appendix–A. Molecular mechanisms of cancer immunotherapies

While the immune system is naturally equipped to detect and attack malignant cells, the myriad of cancer immune evasion decreases their anti-pathogenic function. Tumor antigens are often weakly immunogenic, resulting in low precursor frequency and T-cell receptor (TCR) affinity of tumor infiltrating lymphocytes (TILs) [1]. Furthermore, tumors can induce a state of immune ignorance, preventing infiltration of cytotoxic T-lymphocytes (CTLs) into the tumor core [2]. Immune suppressive cells, such as regulatory T-cells (Tregs) and pro-inflammatory myeloid related suppressor cells, can also be recruited and expanded by tumors *in situ* through the generation of chemokine gradients and the secretion of stimulatory factors such as TGF-ß [3]. Additionally, oncogenes and aberrant signaling pathways induce secretion of factors that promote survival and differentiation of specific immune infiltrates such as tumor associated macrophages (TAMs). Through paracrine feedback loops, these immune populations will secrete the specific chemokines and cytokines necessary to generate an immunosuppressive tumor microenvironment (TME) with promotion of tumor angiogenesis, growth, and metastasis [2]. In the case of chronic exposure, as in the TME, T-cells differentiate into an "exhausted" state of dysfunction. Exhausted TILs are marked by upregulation of inhibitory receptors, decreased effector function and memory formation, and physical deletion. In turn, cancerous cells overexpress ligands of inhibitory receptors [1].

A whole suite of therapeutics—immune checkpoint inhibitors (ICIs), cell therapies, and vaccines—meant to enhance anti-tumor immunity and counteract tumor escape is at various stages of development. **Table 1** provides examples of several therapeutics, including their targets, and mechanisms of action. This includes seven ICIs and two chimeric antigen receptor T-cells (CAR-T) approved by the FDA [4]. The two most clinically successful targets of ICIs are programmed cell death protein 1 (*PD-1*) and cytotoxic T-cell lymphocyte antigen-4 (*CTLA-4*). ICIs block ligand engagement of a broad class of inhibitory receptors expressed by immune cells. When the inhibitory receptors are engaged by their ligands, they repress activating signals and anti-pathogenic functions of the immune system, e.g., phagocytosis [5]. These inhibitory receptors (i.e., immune checkpoints) were originally identified via a shared inhibitory motif – immunoreceptor tyrosine-based inhibitory motifs (ITIM), which recruits phosphatases (SHP1 and SHP2) containing Src homology 2 domain. However, recent studies found that these inhibitory receptors can exert similar inhibitory functions independent of ITIM motifs [6]. Essentially, the immune system evolved these checkpoints to regulate immune activation, and such regulatory mechanisms are critical in maintaining self-tolerance and preventing autoimmunity. For instance, *CTLA-4* and *PD-1* can engage with *B7-1*/*B7-2* (*CD80*/*CD86*) and *PD-L1*/*PD-L2*, respectively, directly inhibiting *CD28* co-stimulation and resulting in reduced T cell proliferative capacity and decreased secretion of cytokines [7,8].

Adoptive cell therapy (ACT) is yet another form of immunotherapy that attempts to enhance the potency and specificity of CTLs. The three major types of ACT are TIL-ACT, TCR-ACT, and CAR-T. The general workflow for generating these therapeutics involves isolating

autologous CTLs from patients' peripheral blood, using genetic engineering tools to modify receptor expression, expanding the population, and reintroducing these cells into the patient. In this review, we will focus on CAR-T, as it is the only ACT with FDA approved products. To create CAR-T therapeutics, T lymphocytes are isolated from patients' peripheral blood and engineered to express an antigen-specific receptor. Specifically, novel membrane proteins with antigen-specific extracellular domains—normally an antibody single chain variable domain (scFv)—as well as co-stimulatory molecules such as CD28 and T-cell activating intracellular domains such as *CD3ζ* are engineered into the cell. These domains are introduced as a single polypeptide, and they bypasses MHC activation requirements of the *TCR* [9]. While the feasibility of MHC-independent activation of T-cells was first demonstrated in 1989 with a CAR specific to 2,4,6-trinitrophenyl, effective CAR-T therapeutics have only recently emerged [10]. There are currently two FDA-approved CAR-T ACTs—Tisagenlecluecel (Kymriah) and Axicabtagene ciloleucel (Yescarta)—that both use membrane expression of scFVs to target *CD19* in B-cell malignancies [11,12]. The two treatments differ in their co-stimulatory molecules—Axicabtagene ciloleucel uses *CD28* whereas Tisagenlecluecel expresses *CD137* (*4-1BB*).

While the above reviewed FDA-approved cancer immunotherapeutics have revolutionized modern cancer treatment, their efficacy is limited to only certain tumors and patients. There remain many challenges associated with optimizing production quality, production efficiency, and efficacy of mAb-based ICIs. To address these challenges, scientists are engaged in a continuous search for novel targets (**Additional file 1: Appendix B**) and new technologies (**Additional file 1: Appendix C**) to enhance potency and overcome tumor evasion in cancer immunotherapies. With regard to the CAR-T ACT, the concomitant life-threatening toxicities are one of the primary issues. For example, on-target, off-tumor reactivity to neoantigens or cross-reactivity to nontarget proteins expressed by normal tissues can cause adverse events. The most prominent CAR-T toxicity is cytokine release syndrome (CRS), caused by the rapid release of cytokines into the bloodstream. Cytokines are either directly produced by CAR-Ts or their production is induced in other immune cell populations via interactions with CAR-T. CRS can cause a wide range of symptoms, such as fever, migraines, pulmonary edema, and colitis and in some cases results in death [13,14]. In parallel, there are barriers associated with production quality, production efficiency, and efficacy of patient-derived CAR-T cells related to graft-versus-host-disease and rejection. As such, various approaches to generate allogeneic CAR T-cells derived from healthy donors are being developed (**Additional file 1: Appendix D**) [15,16]. While valuable insights have been gained, much is still unknown about how glycosylation modulate immunotherapy efficacy and whether this knowledge can be exploited to develop better cancer immunotherapies, indicating that there is significant room for improvement.

Appendix–B. Novel targets to overcome tumor evasion

B.1 Novel ICI targets

ICIs were developed to block inhibitory receptors and enhance anti-tumor immunity by preventing downstream signaling and recovering exhausted T-cells [17]. However, as a result of the limited proportion of patients responsive to CTLA-4 and PD-1 treatment, in part due to compensatory co-inhibitory pathways to blockade and heterogeneity of the tumor microenvironment, several other potential targets are currently being investigated. These include (1) LAG-3, which inhibits CD4+ T-cells via engagement of major histocompatibility complex (MHC) class II stably bound to peptide [18,19]. LAG-3 has also been implicated in regulation of CD8+ T-cells [20,21]. (2) Tim-3, which is constitutively expressed on myeloid cells, on activated CD4+ Th1 T-cell and Th17 T-cell subsets [22], and activated CD8+ T-cells [23]. Blockade of Tim-3 can rescue anti-pathogenic function of exhausted T-cells in chronic infection [24,25]. There are four known ligands of TIM-3: galectin-9, HMGB1, PtdSer, and Ceacam-1, which can act both in *trans* or in *cis* via a self-regulatory mechanism [26]. Importantly, Tim-3 can also play a context-dependent activating role in non-exhausted T-cells and in enabling cross-presentation by dendritic cells (DCs) [23,27]. (3) V-domain Ig-containing Suppressor of T cell Activation (VISTA) can function similarly to other inhibitory receptors described, but can also exert inhibitory influence as a ligand to T-cells in *trans* and may further suppress myeloid activity [28]. (4) TIGIT represses T-cell activation via the co-stimulatory receptor CD226 in a manner analogous to CTLA-4/CD28 signaling via competitive binding of ligands nectin-like proteins necl-5 (CD155) and nectin-2 (CD112) [29]. Finally, (5) the TAM receptor tyrosine kinases (TYRO3, AXL, and MERTK), with growth-arrest-specific 6 (GAS6) and ligands protein S (PROS1), are potential targets due to their negative regulation of DC activity [30].

B.2 Novel co-stimulatory targets

In contrast to ICIs, therapeutics that act as agonists of co-stimulatory molecules on immune cells are also of interest to immuno-oncologists. One such co-stimulatory receptor is the tumor necrosis factor receptor family–OX40 (CD134), which is expressed by tumor infiltrating lymphocytes (TILs). OX40's cognate ligand is OX40L, which is endogenously expressed on various antigen presenting cells (APCs). Engagement of OX40 by agonistic antibodies increases survival of effector T-cell subsets [31]. It is important to note that OX40 faces certain limitations; some OX40 agonists have activated the immunosuppressive activities of regulatory T-cells (Tregs) and induced antibody-dependent cell cytotoxicity (ADCC) or phagocytosis (ADCP) of effector T-cells [32]. Conversely, OX40 can prevent the generation and functionality of Tregs [31]. Other co-stimulatory targets include (1) inducible co-stimulator (ICOS), a receptor specific to T-cells—usually CD4+ T-cells. ICOS engagement promotes biosynthesis of molecules that mediate cell-cell interactions, proliferation, and lymphokine secretion [33]. (2) Glucocorticoid-induced TNF

receptor family-related protein (GITR) is upregulated on TILs and has cognate ligand GITRL expressed on APCs and epithelial cells [34]. (3) 4-1BB, while expressed at comparable levels in CD4+ and CD8+ T-cells, demonstrates a bias in co-stimulatory signaling for CD8+ T-cells and also promotes survival [19].

Appendix–C. Novel technologies to overcome tumor evasion

In addition to identifying novel immune checkpoint targets, researchers have attempted combinatorial administration of known targets to improve efficacy of therapeutics. For example, combining nivolumab (Opdivo) and ipilimumab (Yervoy), which target PD-1 and CTLA-4 respectively, demonstrated increased rates of objective response in patients with melanoma relative to either treatment alone [35]. This makes sense, considering that the two treatments target different mechanisms of action and distinct subsets of exhausted CD8+ T-cells, and that anti-CTLA-4 also targets CD4+ T-cells [36]. Positive results have also been demonstrated for combinations targeting PD-1 and CD20 in follicular lymphoma [37], PD-1 and LAG-3 in ovarian cancer [38,39], PD-1 with TIM-3 [40], immune checkpoint targets combined with other approaches such as chemotherapy and epigenetic targets [41]. Additionally, researchers have explored alternatives to immune checkpoint therapeutics, such as neo-antigen vaccines. While there is no FDA-approved vaccine released as of yet, there are several studies at the preclinical stage. Tumor infiltrating lymphocytes (TILs) can be specific either to antigens expressed on a restricted number of tissue types and tumors or to neo-antigens, i.e., antigens expressed exclusively by the tumor. Neo-antigens, unlike non-specific antigens, circumvent the issue of weak immunogenicity induced by central thymic tolerance. Furthermore, these vaccines may be better equipped to treat cancers with a high mutational burden. Personalized vaccines for neo-antigen-specific T-cells are generated by whole exome sequencing to detect somatic mutations, subsequent HLA binding peptide prediction, and synthesis and pooling of various combination of these peptides [42]. A recent phase I clinical study of personalized neo-antigen vaccination was conducted against stage IIIB/C and IVM1a/b cutaneous melanoma in combination with anti-PD-1 pembrolizumab for patients that experienced recurrence. This study reported promising results at ~25 months after vaccination, with four out of six patients indicating no recurrence and the remaining two demonstrating complete radiographic response after treatment with pembrolizumab (https://clinicaltrials.gov/ct2/show/NCT01970358) [43].

Appendix–D. Novel technologies to overcome graft-versus-host-disease

A major challenge to CAR-T ACT is the resultant life-threatening toxicities, such as the cytokine release syndrome (CRS) [14] and the barriers associated with production quality, production efficiency, and efficacy of patient-derived CAR-T cells. At present, various approaches are being developed to manufacture allogeneic CAR T-cells derived from healthy donors [15,16]. The two major challenges to generating allogeneic CAR T-cells are concerns of graft-versus-host-disease that host tissue could be attacked by donor immune cells due to the TCR recognizing human leukocyte antigen (HLA) mismatch, and the inverse issue of rejection, in which the host immune system attacks the donor therapeutic cells. Yet, patient-derived, autologous CAR T-cells have a higher probability of dysfunction, and there are issues with consistently and successfully engineering them in time for treatment. Recently, CYAD-101, which expresses a multi-antigen specific NKG2D CAR and aims to treat colorectal cancer, is the first to enter clinical trials (https://clinicaltrials.gov/ct2/show/NCT03692429). CYAD-101 prevents graft versus host disease by co-expression of a TCR inhibitory molecule (TIM) which inhibits TCR signaling and hence possible alloreactivity.

Appendix–E. The coming age of Systems Glycobiology in cancer research

Figure S1 shows many advanced developments in cancer immunology (Nobel Prize or Milestone of cancer immunology (blue colors)) in the past decades. Many of these developments are already translated into new medicines as cancer immunotherapies (the FDA approved cancer immunotherapies (red colors)). In parallel, advanced developments in systematic modelling of glycosylation machinery (purple colors) and the analytical methods and computational tool for study glycan epitopes (green colors) have greatly enhance our understanding on the complex glycosylation machinery. In the next decades, it is expected that we can apply Systems Glycobiology in cancer research to overcome many current challenges in cancer immunotherapy. It should be noted that we only selected several Nobel Prize or Milestone of cancer immunology in this figure due to limited space. This figure did not completely include all the Nobel prizes relevant to cancer immunotherapies. For example, the Nobel prize awarded for the development of monoclonal antibody technology (Jerne, Köhler and Milstein, Physiology/Medicine, 1984), and the Nobel prizes awarded for the discovery of cellular cytotoxicity by T cells (Doherty and Zinkernagel, Physiology/Medicine, 1996). Also, several Nobel prizes relevant to glycobiology (e.g., discovery of human blood groups, Landsteiner, Physiology/Medicine, 1930; discovery of sugar nucleotides, Leloir, Chemistry, 1970) or the technologies that enabled systematic analysis of glycans and glycosylation machinery (e.g., development of soft ionization technique for MS, by Fenn and Tanaka, Chemistry, 2002; discovery and application of CRISPR/Cas9, by Doudna and Charpentier, Chemistry, 2020).

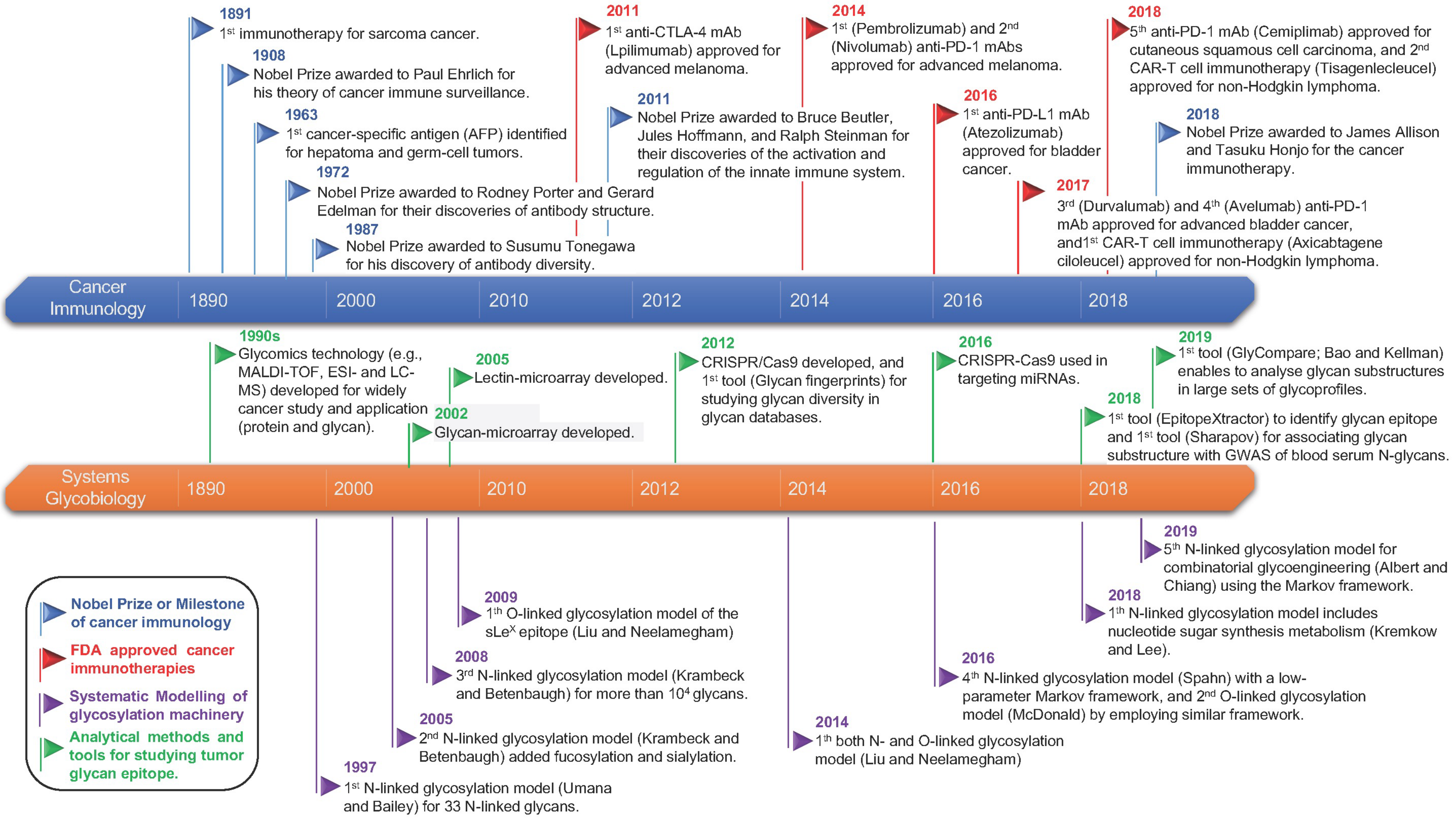

Figure S1. The coming age of Systems Glycobiology in cancer research. (**Top panel**) Timeline of Nobel Prize or Milestone of cancer immunology (blue colors) and the FDA approved cancer immunotherapies (red colors). (**Bottom panel**) Timeline of systematic modelling of glycosylation machinery (purple colors) and the analytical methods and computational tool for study glycan epitopes (green colors).

References

1. Jiang Y, Li Y, Zhu B. T-cell exhaustion in the tumor microenvironment. Cell Death & Disease. 2015;6:e1792–e1792.

2. Binnewies M, Roberts EW, Kersten K, Chan V, Fearon DF, Merad M, et al. Understanding the tumor immune microenvironment (TIME) for effective therapy. Nature Medicine. 2018;24:541–50.

3. Becht E, de Reyniès A, Giraldo NA, Pilati C, Buttard B, Lacroix L, et al. Immune and Stromal Classification of Colorectal Cancer Is Associated with Molecular Subtypes and Relevant for Precision Immunotherapy. Clinical Cancer Research. 2016;22:4057–66.

4. Li Z, Song W, Rubinstein M, Liu D. Recent updates in cancer immunotherapy: a comprehensive review and perspective of the 2018 China Cancer Immunotherapy Workshop in Beijing. Journal of hematology & oncology. 2018;11:142.

5. Ravetch J V, Lanier LL. Immune inhibitory receptors. Science. 2000;290:84–9.

6. De Sousa Linhares A, Leitner J, Grabmeier-Pfistershammer K, Steinberger P. Not All Immune Checkpoints Are Created Equal. Frontiers in Immunology. 2018;9:1–15.

7. Schneider H, Downey J, Smith A, Zinselmeyer BH, Rush C, Brewer JM, et al. Reversal of the TCR stop signal by CTLA-4. Science. 2006;313:1972–5.

8. Hui E, Cheung J, Zhu J, Su X, Taylor MJ, Wallweber HA, et al. T cell costimulatory receptor CD28 is a primary target for PD-1-mediated inhibition. Science. 2017;355:1428–33.

9. June CH, O'Connor RS, Kawalekar OU, Ghassemi S, Milone MC. CAR T cell immunotherapy for human cancer. Science. 2018;359:1361–5.

10. Gross G, Waks T, Eshhar Z. Expression of immunoglobulin-T-cell receptor chimeric molecules as functional receptors with antibody-type specificity. Proceedings of the National Academy of Sciences of the United States of America. 1989;86:10024–8.

11. Neelapu SS, Locke FL, Bartlett NL, Lekakis LJ, Miklos DB, Jacobson CA, et al. Axicabtagene Ciloleucel CAR T-Cell Therapy in Refractory Large B-Cell Lymphoma. New England Journal of Medicine. 2017;377:2531–44.

12. Maude SL, Laetsch TW, Buechner J, Rives S, Boyer M, Bittencourt H, et al. Tisagenlecleucel in Children and Young Adults with B-Cell Lymphoblastic Leukemia. New England Journal of Medicine. 2018;378:439–48.

13. Morgan RA, Yang JC, Kitano M, Dudley ME, Laurencot CM, Rosenberg SA. Case Report of a Serious Adverse Event Following the Administration of T Cells Transduced With a Chimeric Antigen Receptor Recognizing ERBB2. Molecular Therapy. 2010;18:843–51.

14. Morgan RA, Chinnasamy N, Abate-Daga D, Gros A, Robbins PF, Zheng Z, et al. Cancer regression and neurological toxicity following anti-MAGE-A3 TCR gene therapy. Journal of immunotherapy. 2013;36:133–51.

15. Eyquem J, Mansilla-Soto J, Giavridis T, van der Stegen SJC, Hamieh M, Cunanan KM, et al. Targeting a CAR to the TRAC locus with CRISPR/Cas9 enhances tumour rejection. Nature. 2017;543:113–7.

16. Graham C, Jozwik A, Pepper A, Benjamin R. Allogeneic CAR-T Cells: More than Ease of Access? Cells. 2018;7:155.

17. Blank C, Brown I, Peterson AC, Spiotto M, Iwai Y, Honjo T, et al. PD-L1/B7H-1 inhibits the effector phase of tumor rejection by T cell receptor (TCR) transgenic CD8+ T cells. Cancer research. 2004;64:1140–

5.

18. Maruhashi T, Okazaki I, Sugiura D, Takahashi S, Maeda TK, Shimizu K, et al. LAG-3 inhibits the activation of CD4+ T cells that recognize stable pMHCII through its conformation-dependent recognition of pMHCII. Nature Immunology. 2018;19:1415–26.

19. Workman CJ, Dugger KJ, Vignali DAA. Cutting Edge: Molecular Analysis of the Negative Regulatory Function of Lymphocyte Activation Gene-3. The Journal of Immunology. 2002;169:5392–5.

20. Grosso JF, Kelleher CC, Harris TJ, Maris CH, Hipkiss EL, De Marzo A, et al. LAG-3 regulates CD8+ T cell accumulation and effector function in murine self- and tumor-tolerance systems. The Journal of clinical investigation. 2007;117:3383–92.

21. Cook KD, Whitmire JK. LAG-3 Confers a Competitive Disadvantage upon Antiviral CD8+ T Cell Responses. Journal of immunology. 2016;197:119–27.

22. Hastings WD, Anderson DE, Kassam N, Koguchi K, Greenfield EA, Kent SC, et al. TIM-3 is expressed on activated human CD4+ T cells and regulates Th1 and Th17 cytokines. European journal of immunology. 2009;39:2492–501.

23. Avery L, Filderman J, Szymczak-Workman AL, Kane LP. Tim-3 co-stimulation promotes short-lived effector T cells, restricts memory precursors, and is dispensable for T cell exhaustion. Proceedings of the National Academy of Sciences. 2018;115:2455–60.

24. Jones RB, Ndhlovu LC, Barbour JD, Sheth PM, Jha AR, Long BR, et al. Tim-3 expression defines a novel population of dysfunctional T cells with highly elevated frequencies in progressive HIV-1 infection. Journal of Experimental Medicine. 2008;205:2763–79.

25. Golden-Mason L, Palmer BE, Kassam N, Townshend-Bulson L, Livingston S, McMahon BJ, et al. Negative Immune Regulator Tim-3 Is Overexpressed on T Cells in Hepatitis C Virus Infection and Its Blockade Rescues Dysfunctional CD4+ and CD8+ T Cells. Journal of Virology. 2009;83:9122–30.

26. Du W, Yang M, Turner A, Xu C, Ferris R, Huang J, et al. TIM-3 as a Target for Cancer Immunotherapy and Mechanisms of Action. International Journal of Molecular Sciences. 2017;18:645.

27. Nakayama M, Akiba H, Takeda K, Kojima Y, Hashiguchi M, Azuma M, et al. Tim-3 mediates phagocytosis of apoptotic cells and cross-presentation. Blood. 2009;113:3821–30.

28. Nowak EC, Lines JL, Varn FS, Deng J, Sarde A, Mabaera R, et al. Immunoregulatory functions of VISTA. Immunological Reviews. 2017;276:66–79.

29. Dougall WC, Kurtulus S, Smyth MJ, Anderson AC. TIGIT and CD96: new checkpoint receptor targets for cancer immunotherapy. Immunological Reviews. 2017;276:112–20.

30. Akalu YT, Rothlin C V, Ghosh S. TAM receptor tyrosine kinases as emerging targets of innate immune checkpoint blockade for cancer therapy. Immunological Reviews. 2017;276:165–77.

31. Aspeslagh S, Postel-Vinay S, Rusakiewicz S, Soria J-C, Zitvogel L, Marabelle A. Rationale for anti-OX40 cancer immunotherapy. European Journal of Cancer. 2016;52:50–66.

32. Turner JG, Rakhmilevich AL, Burdelya L, Neal Z, Imboden M, Sondel PM, et al. Anti-CD40 Antibody Induces Antitumor and Antimetastatic Effects: The Role of NK Cells. The Journal of Immunology. 2001;166:89–94.

33. Hutloff A, Dittrich AM, Beier KC, Eljaschewitsch B, Kraft R, Anagnostopoulos I, et al. ICOS is an inducible T-cell co-stimulator structurally and functionally related to CD28. Nature. 1999;397:263–6.

34. Marin-Acevedo JA, Dholaria B, Soyano AE, Knutson KL, Chumsri S, Lou Y. Next generation of immune checkpoint therapy in cancer: new developments and challenges. Journal of Hematology & Oncology. Journal of Hematology & Oncology; 2018;11:39.

35. Wolchok JD, Kluger H, Callahan MK, Postow MA, Rizvi NA, Lesokhin AM, et al. Nivolumab plus Ipilimumab in Advanced Melanoma. New England Journal of Medicine. 2013;369:122–33.

36. Wei SC, Levine JH, Cogdill AP, Zhao Y, Anang N-AAS, Andrews MC, et al. Distinct Cellular Mechanisms Underlie Anti-CTLA-4 and Anti-PD-1 Checkpoint Blockade. Cell. 2017;170:1120-1133.e17.

37. Westin JR, Chu F, Zhang M, Fayad LE, Kwak LW, Fowler N, et al. Safety and activity of PD1 blockade by pidilizumab in combination with rituximab in patients with relapsed follicular lymphoma: a single group, open-label, phase 2 trial. The Lancet Oncology. 2014;15:69–77.

38. Matsuzaki J, Gnjatic S, Mhawech-Fauceglia P, Beck A, Miller A, Tsuji T, et al. Tumor-infiltrating NY-ESO-1–specific CD8 + T cells are negatively regulated by LAG-3 and PD-1 in human ovarian cancer. Proceedings of the National Academy of Sciences. 2010;107:7875–80.

39. Huang R, Eppolito C, Lele S, Shrikant P, Matsuzaki J, Odunsi K. LAG3 and PD1 co-inhibitory molecules collaborate to limit CD8+ T cell signaling and dampen antitumor immunity in a murine ovarian cancer model. Oncotarget. 2015;6:27359–77.

40. Sakuishi K, Apetoh L, Sullivan JM, Blazar BR, Kuchroo VK, Anderson AC. Targeting Tim-3 and PD-1 pathways to reverse T cell exhaustion and restore anti-tumor immunity. Journal of Experimental Medicine. 2010;207:2187–94.

41. J. Harris S, Brown J, Lopez J, A. Yap T, J. Harris S, Brown J, et al. Immuno-oncology combinations: raising the tail of the survival curve. Cancer Biology & Medicine. 2016;13:171–93.

42. Schumacher TN, Schreiber RD. Neoantigens in cancer immunotherapy. Science. 2015;348:69–74.

43. Ott PA, Hu Z, Keskin DB, Shukla SA, Sun J, Bozym DJ, et al. An immunogenic personal neoantigen vaccine for patients with melanoma. Nature. 2017;547:217–21.
